# New animal model of chronic gout reproduces pathological features of the disease in humans

**DOI:** 10.1136/rmdopen-2023-003499

**Published:** 2023-11-16

**Authors:** Jiwei Wang, Peiqi Hao, Xianrun Sun, Richard Ward, Tao Tang, Xi Chen, Yihong Liu, Guancong Luo, Yang Yang, Cheng Xiang, Su An, Tian-Rui Xu

**Affiliations:** 1 Faculty of Life Science and Technology, Kunming University of Science and Technology, Kunming, Yunnan, China; 2 Department of Orthopedic Surgery, Affiliated Hospital of Kunming University of Science and Technology, Kunming, Yunnan, China; 3 Centre for Translational Pharmacology, Institute of Molecular Cell and Systems Biology, College of Medical, Veterinary and Life Sciences, University of Glasgow, Glasgow, UK; 4 Medical School, Kunming University of Science and Technology, Kunming, Yunnan, China

**Keywords:** Gout, Arthritis, Experimental, Antirheumatic Agents

## Abstract

**Objectives:**

Gout, as the most prevalent form of inflammatory arthritis, necessitates the use of animal models to investigate the molecular mechanisms involved in its development. Therefore, our objective was to develop a novel chronic mouse model of gout that more closely mimics the progression of gout in humans.

**Methods:**

A novel chronic mouse model of gout was established by a simple method, which does not require high technical proficiency, predominantly involves daily intraperitoneal injections of potassium oxonate for approximately 4 months, combined with a high fat-diet and injections of acetic acid into the hind paws to facilitate the formation of monosodium urate (MSU). Arthritis scores and paw oedema were assessed, behavioural tests were conducted, and histopathological and imaging evaluations of the arthritic paw joints were performed.

**Results:**

After 4 months of induction, mice in the model group exhibited noticeable increases in arthritis severity, joint and cartilage damage, as well as bone erosion. Gomori’s methenamine silver stain revealed the presence of MSU crystal deposition or tophi in the paw joints or ankle joints of up to 37.9% of the model mice (11 out of 29 mice). Moreover, treatment with benzbromarone effectively prevented the further development of gout or tophi formation in model mice.

**Conclusions:**

Our model more accurately replicates the pathological features of gouty arthritis compared with gout induced by MSU crystal injections. Therefore, it is particularly suitable for further investigations into the pathogenesis of gout and also serves as a valuable platform for screening potential antigout agents.

**WHAT IS ALREADY KNOWN ON THIS TOPIC**

- Several animal models of gouty arthritis have been explored, involving the direct injection of monosodium urate crystals (MSU) into joint locations. However, current gout models fail to replicate the chronic deposition of MSU observed in human gout, which is a key pathological feature.

**WHAT THIS STUDY ADDS**

- A novel mouse model of gout was established by repeated daily injections of a low dose of potassium oxonate to moderately increase the serum uric acid (sUA) levels and avoid severe hepatic and renal dysfunction as well as mouse mortality caused by sustained high levels of sUA before the development of gouty arthritis, combining with a high fat-diet and the injection of 0.1% acetic acid into the hind paws to facilitate the formation of MSU crystals.
- The development of gouty arthritis in a mouse model promoted by a high-fat diet may be attributed to the fact that increased adiposity is a significant risk factor for gout.
- The injection of acetic acid may facilitate the formation of MSU crystals by lowering the pH value of the joint microenvironment, disrupting the integrity of the joint structure and exposing cartilage components.

**HOW THIS STUDY MIGHT AFFECT RESEARCH, PRACTICE OR POLICY**

- A chronic mouse model established in this study accurately replicated the clinical and pathological features observed in human gout, making it highly suitable for further investigations into the pathogenesis of gouty arthritis. Moreover, this model holds great potential as an ideal screening platform for testing antigout agents.

## Introduction

Gout is the most prevalent form of inflammatory arthritis^1 2^ and is caused by chronically elevated serum uric acid (sUA) levels above the saturation point for monosodium urate (MSU) crystal formation.^3^ The deposition of MSU crystals is predominantly to be found in the peripheral joints and surrounding tissues. The two major factors promoting MSU crystal formation are chronic hyperuricaemia and local tissue characteristics including cartilage damage and declining pH of the synovial fluid that facilitate MSU crystal nucleation and growth.^4^ Hyperuricaemia is well known to be the most important risk factor for the development of gout, MSU crystals can form when the sUA level rises above the saturation threshold for MSU crystal formation. It is worth noting that local factors have been considered to play an important role in crystal formation, such as mechanical stress, damage to cartilage and its components, and pH of synovial fluid. Mechanical trauma or cartilage damage can lead to the exposure of urate to synovial fluid and cartilage elements which have been shown to play an important role in the formation of MSU crystal.^4 5^ Similarly, the presence of an acidic environment has also been shown to promote MSU crystallisation and also declining pH levels have been detected in the synovial fluid of acute gout.^4^ Additionally, hyperuricaemia is recognised as a new marker of metabolic syndrome.^6 7^ Epidemiological investigation results have indicated a close relationship between sUA levels and the development of metabolic syndrome among children as well as adults,^8–12^ furthermore higher adiposity is a strong risk factor for gout in men.^13 14^

Mouse models of gout are indispensable tools for the investigation of the cellular and molecular mechanisms underlying the development of gout. In the past, several animal models of crystal-induced acute inflammatory response have been studied through direct injection of MSU crystals into articular locations or air pouches.^15 16^ However, the current models of gout do not reproduce the features of gout that are characterised by the chronic development of crystal deposition, cartilage damage, neutrophil infiltration, bone erosion and tophi formation.

In order to mimic the development of human gout or gouty arthritis more closely, we present here a novel mouse model of gout induced by repeated daily injections of a low dose of potassium oxonate to moderately increase the sUA levels, combined with a high fat-diet and the injection of 0.1% acetic acid into the hind paws of mice to facilitate the formation of MSU crystals. Arthritis score and paw oedema were measured, behavioural tests were carried out and the histopathological and imaging assessment of arthritic paw joints was performed. We also assessed the expression of IL-1β, IL-6, TNF-α and IL-10 in the paw joints using ELISA, qRT-PCR and immunohistochemical staining. This chronic mouse model of gouty arthritis provides a preclinical model to test therapeutics and will be instrumental in further exploring the molecular and cellular mechanisms underlying gouty arthritis.

### Material and methods

### Animal studies

All experiments were conducted in accordance with the methods of the UK Animals (Scientific Procedures) Act 1986 and our institutional guidelines for the care and use of animals. Male Kunming mice were purchased from Shanghai Lingchang Biotechnology (Shanghai, China), and housed in a room at a constant temperature of 22°C±2°C and humidity of 55%±19% under a 12-hour light/12-hour dark cycle and allowed free access to food and water. To induce gouty arthritis, 6-week-old mice were given daily intraperitoneal injections of a low concentration of potassium oxonate (150 mg/kg body weight), in combination with the injection of 20 µL 0.1% of acetic acid into both hind paws with a microliter syringe twice a week and feeding a high-fat diet (60% fat diet). One hundred and twenty mice were randomly divided into two groups: group 1 (n=40) served as the control group, which were fed a high-fat diet and received injections of 20 µL 0.1% of acetic acid every 3 days; group 2 (n=80) served as the model group, which was administered daily i.p. injections of a low dose of potassium oxonate (150 mg/kg body weight) in addition to a high-fat diet and injections of acetic acid. Two months later, group 2 was further divided into two subgroups, group 2–1 and group 2–2, with induction conditions unchanged. Group 2–2 received oral administration of benzbromarone (20 mg/kg/day) for about 2 months, while group 2–1 was treated with saline in exactly the same way, and served as negative control of the benzbromarone group.

### Measurement of sUA and plasma glucose levels

Whole blood samples were collected and allowed to clot for 30 min at room temperature and then centrifuged to obtain the serum. The serum samples were stored at −20°C until assayed. The sUA level was determined by using a previously reported high performance liquid chromatography (HPLC) method.^17^ Plasma glucose levels were analysed using the OneTouch UltraMini meter (LifeScan, California, USA).

### Assessment of arthritis severity and behavioural testing in mice

The assessment of the severity of arthritis and the behavioural tests were conducted in a blinded manner and measured at least 1 week after injections of acetic acid to eliminate its effects on testing results.

For the determination of paw volume, paw swelling (oedema) was measured by a water displacement plethysmometer (Ugo Basile, Comerio, Italy). The plethysmometer is a micrcontrolled volume metre specially designed for the accurate measurement of mouse paw swelling.^18^ Clinical assessment of gouty arthritis was determined as follows: 0, normal, no oedema or swelling; (1) slight oedema and limited erythema; (2) slight oedema and erythema from the digits to the ankles; (3) moderate oedema and erythema from the digits to the ankles; (4) complete swelling and erythema of the entire paw and an inability to bend the ankles. Scores were added for both hind paws to a maximal composite score of 8.

To evaluate thermal pain sensitivity, a mouse was placed on top of a hot plate apparatus that was preset to 52°C and the time taken to respond (as distinguished by flicking or licking the hind paws) was noted and the mouse was removed from the hot plate. To avoid injury to the mice, a maximum of 60 s was set.

To test the ability to hang by the hind paws, a mouse was placed on a cage lid which was then turned upside down, the suspended mice should hold on to the grid with its paws to avoid falling. In this test, each hang period must begin with all four paws of the mouse grasping the grid. Shortly before the fall, mice usually lose their grasp of the grid one or two hind paws, then we stop the stopwatch. For thermal pain sensitivity and hanging ability tests described above, two trials were done to ensure the mice were familiarised with the testing condition and then three trials were done at 10 min intervals to allow a recovery period. The data were expressed as an average of three trials.

### Histopathology examination

For histological analyses, the hind paws and ankle regions were dissected and fixed overnight in 10% formalin and then decalcified in 14% EDTA until bones were pliable, embedded in paraffin and sectioned at 4–5 µm. Tissue sections were stained with H&E stain, and were evaluated in a blinded fashion and photographed under a light microscope (Olympus BX60, Tokyo, Japan). The degree of inflammation, synovial proliferation, bone erosions and cartilage damage were scored, as described by Pettit *et al*.^19^ Briefly, they were scored as follows: 0, normal; (1) minimal; (2) mild; (3) moderate; (4) marked and (5) severe. Cartilage destruction was determined by Safranin O-Fast Green Staining, and the International Osteoarthritis Research Society International (OARSI) scoring system was used to evaluate the degree of cartilage damage.^20^ For renal histopathological analysis, 4 µm thick kidney sections embedded in paraffin were cut and mounted on glass slides, and then stained with H&E for histopathological examination.

### Gomori’s methenamine silver for urate crystals

Gomori’s methenamine silver stain is a classical method for the staining of urate crystals. The deposition of urate crystals was stained according to a modified version of Gomori’s method.^21^ Briefly, the hind paws of mice were fixed in absolute alcohol at 4°C overnight, embedded in paraffin and sectioned at 4–5 µm, deparaffinised with xylene and rinsed with 3 changes of absolute alcohol, and then stained with preheated working methenamine silver for 30 min at 60°C, toned with gold chloride and counterstained with eosin solution. Urates are stained black in a pink-light red background.

### Radiologic imaging

After the mice were euthanised, their hind paws were immediately collected and stored at −80°C. The paws (from the tip of toes to ankle joints) were scanned using an X-ray CT system (Latheta LCT-200, Hitachi Aloka Medical, Tokyo, Japan). The X-ray tube voltage was 50 kVp, the current was constant at 500 μA, and an integration time 3.6 ms was used, with no filter. The axial field of view was 24 mm with an isotropic voxel size of 24 µm. Projection images were reconstructed into three-dimensional images using the VGStudio MAX V.2.1 software (Nihon Visual Science, Tokyo, Japan). The following three-dimensional trabecular bone parameters were obtained: trabecular number (Tb.N), trabecular thickness (Tb.Th) and trabecular separation (Tb.Sp). The severity of bone destruction in three-dimensional micro-CT reconstruction images was assessed, blind, by two osteologists according to the following criteria: 0=normal, no signs of bone erosion/deformity; 1=mild roughness of bone surface; 2=moderate roughness of bone surface and bone deformity; 3=marked roughness of bone surface and signs of bone fusion; 4=whole bone deformity and complete bone fusion.^22^ Bone erosion and joint damage were also evaluated by plain radiography imaging using the Latheta LCT-200 in general radiography mode. Radiographs were analysed by two independent musculoskeletal clinicians in a blinded manner using a modified version of the Larsen score: 0=intact bony outlines and normal joint spaces; 1=slight abnormality of one or two metatarsal bones showing slight bone erosion; 2=obvious early abnormality of the metatarsal or tarsal bones showing bone erosion; 3=medium destructive abnormality of the metatarsal or tarsal bones showing obvious bone erosion; 4=severe destructive abnormality of all metatarsal or tarsal bones, some tarsometatarsal joints were completely eroded and bony joint outlines were partly preserved; 5=mutilating changes and no bony outlines could be deciphered.

### Statistical analysis

All quantitative data were presented as the mean±SD and data were analysed using GraphPad Prism V.9 (GraphPad Software, San Diego, California, USA). Statistically significant differences of arthritis score, paw volume, paw withdraw latency, hanging time, parameters in micro-CT imaging, semiquantitative histopathological and immunohistochemical examination between different groups were assessed using an unpaired two-tailed Student’s t-test. Statistical differences with a probability of p<0.05 are indicated with *, p<0.01 with **, p<0.001 with *** and p<0.0001 with ****.

A full description of immunohistochemical analysis, cytokine ELISA, quantitative real-time PCR, western blot analysis and in vivo lucigenin bioluminescence imaging of nicotinamide adenine dinucleotide phosphate(NADPH) oxidase activity is available in online supplemental material.

10.1136/rmdopen-2023-003499.supp1 Supplementary data

## Results

### Arthritis score, paw oedema and behavioural testing in hyperuricaemia-induced gout mouse model

**Figure 1:**
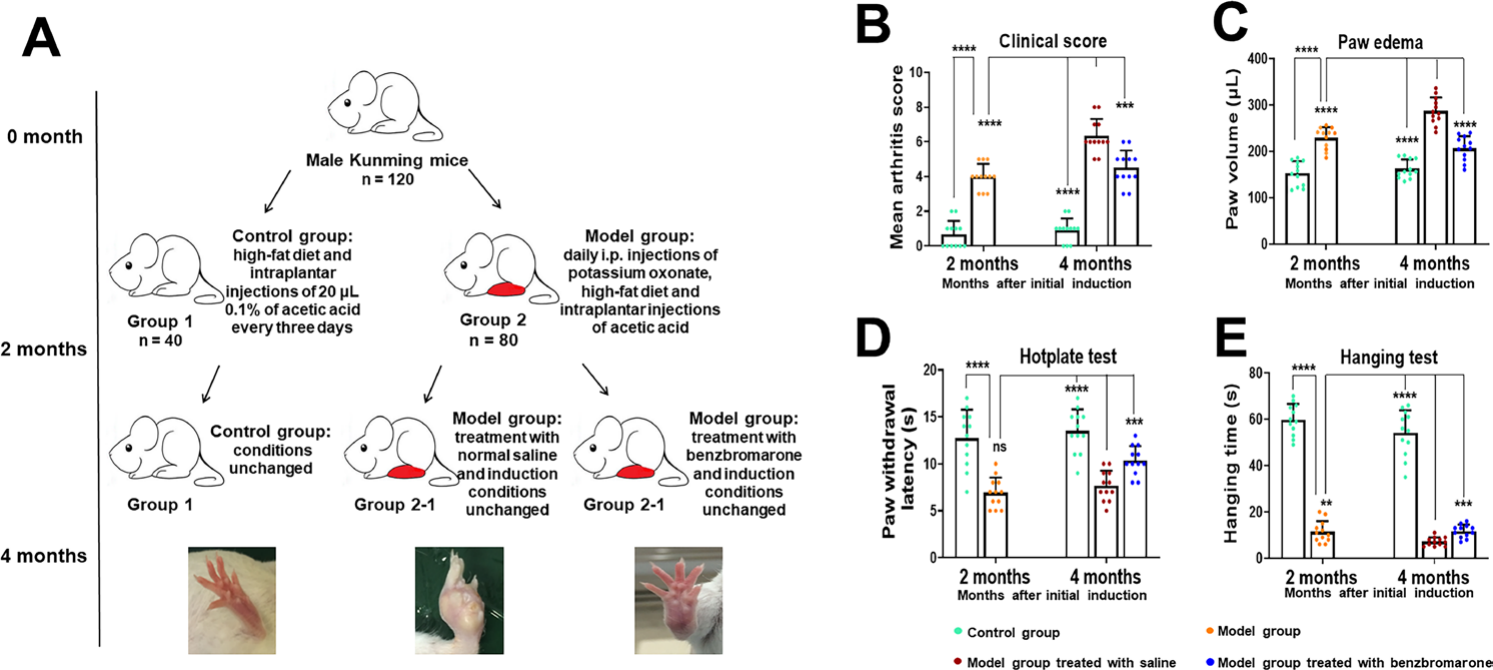
Arthritis score, paw oedema and behavioural testing in the mouse model of gouty arthritis. (A) Schematic diagram of the study protocol. (B–E) The severity of gouty arthritis and functional impairment of paw joints were assessed by clinical scores, measurement of paw swelling, hotplate test and hanging test after 2 and 4 month arthritis induction. (B) The severity of arthritis was scored by two independent observers who were blind to the animal’s treatment. Score system was defined in materials and method section. (C) Oedema in hind paws following the induction of gouty arthritis, as measured by paw volume. (D) Baseline pain and inflammatory pain for heat sensitivity. (E) Hanging test performance of control group, model mice and model mice treated with benzbromarone. Data are represented as the mean±SD, n=12 animals per group. **p<0.01, ***p<0.001, ****p<0.0001 were calculated using the Student’s t-test.

After 2 months of induction with hyperuricaemia in combination with high-fat diet and intraplantar injection of acetic acid, macroscopic evidence of gouty arthritis such as swelling and erythema was clearly observed in model mice (figure 1A–C). Then the model group (group 2) was divided into two subgroups, group 2–1 and group 2–2, with induction conditions unchanged. The group 2–1 and group 2–2 were treated with either saline or benzbromarone for about 2 months, respectively. As shown in figure 1B,C, both the mean arthritis score (p<0.0001) and paw oedema (p<0.0001) in the benzbromarone-treated mice were significantly lower than in saline-treated mice, the reduction of arthritic severity by benzbromarone was remarkable. Both paw and ankle joints in the model mice treated with saline had a more serious swelling and even presented with subcutaneous gouty tophi (figure 1A–C).

Nociceptive sensitivity caused by gouty arthritis was assessed by the hot plate test. This thermal hyperalgesia was significantly enhanced in both the model mice and the benzbromarone-treated mice compared with the control group. Moreover, benzbromarone treatment significantly reduced thermal hyperalgesia in model mice treated with benzbromarone in comparison with model mice treated with saline (p<0.0001) (figure 1D). Additionally, we also investigated the ability to hang by the hind paws in the model mice and controls. These analyses revealed the expected gouty arthritis-related reduction in the hanging ability of the model mice and suggested no significant improvement in the condition of the benzbromarone-treated mice relative to model mice (p<0.0001) (figure 1E). The intraplantar injection of acetic acid was stopped at least 1 week before the above measurements to eliminate its effects on the results.

As shown in online supplemental figures S1–4, intraperitoneal injections of potassium oxonate increased the sUA levels in a dose-dependent manner (online supplemental figure S1A), when compared with the control group, the sUA (online supplemental figure S1B) and plasma glucose levels (online supplemental figure S2) of model mice were significantly elevated at 2 months and 4 months after the initial induction of gouty arthritis. Strikingly, the UA levels in the urine of the model mice significantly decreased in comparison with the control group. This phenomenon may be attributed to the fact that potassium oxonate can increase both the water intake and 24-hour urine volume of mice. Although benzbromarone can raise UA levels in urine, they still did not reach the baseline level (online supplemental figure S1C). The reduction of serum urate levels was observed in benzbromarone-treated model mice (online supplemental figure S1B), while benzbromarone had no obvious effect on the concentrations of plasma glucose (online supplemental figure S2). Moreover, no significant body weight changes were found between the groups (online supplemental figure S3), and no obvious renal pathological damage was observed in model mice (online supplemental figure S4).

### Radiologic imaging and analysis of gouty arthritis progression

**Figure 2:**
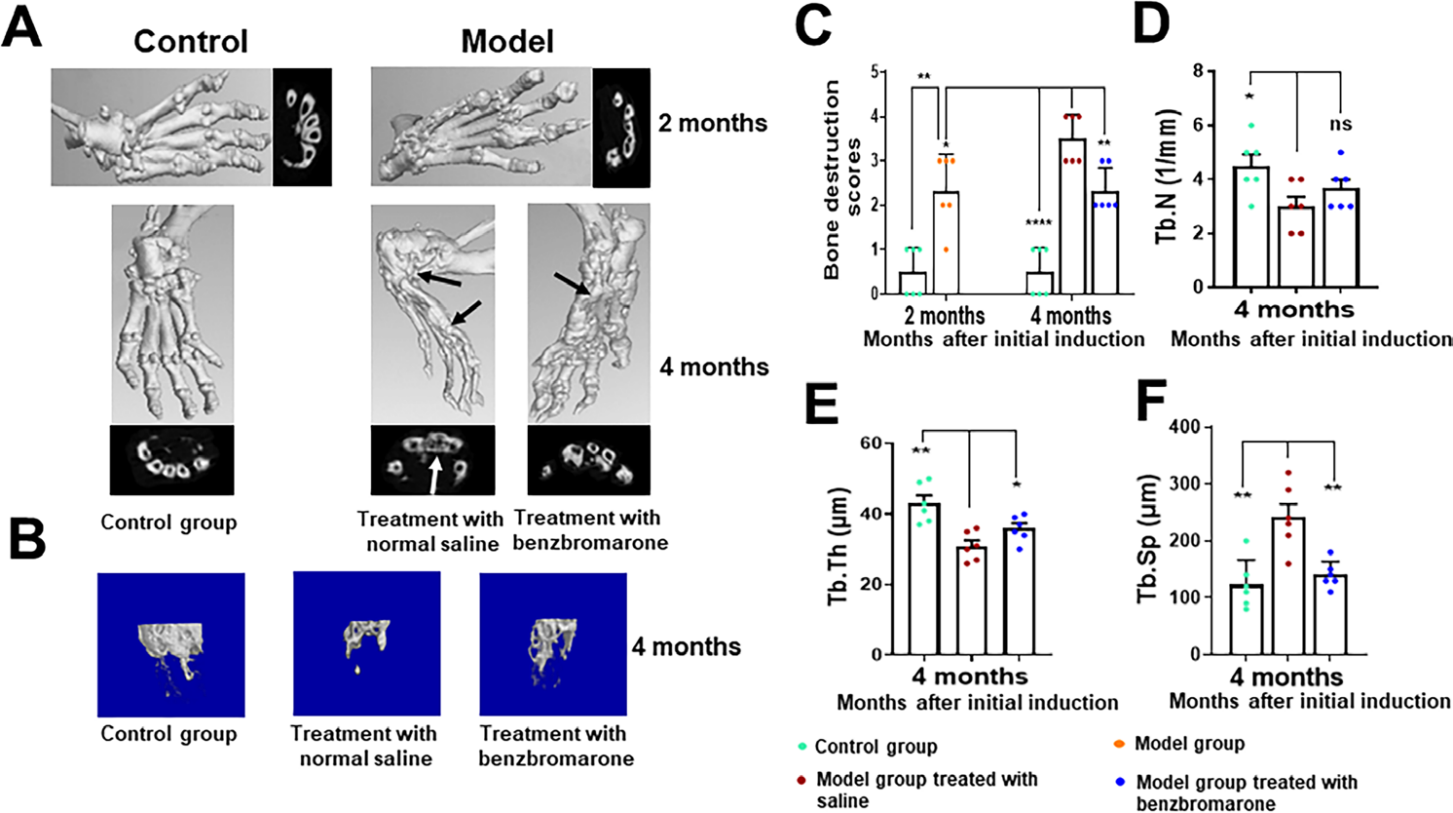
In vivo imaging and quantitative analysis of the progression of gouty arthritis by micro-CT. (A–B) Representative of three-dimensional reconstructions of the bone architecture of hind paws (A) and distal tibial bones (B) in non-induced control mice and hyperuricaemia-induced gouty arthritis model mice treated with vehicle or 20 mg/kg/day benzbromarone. (C) Quantification of bone destruction scores which were attributed from the micro-CT analysis. Data are represented as the mean±SD, (n=6 mice per group). *p<0.05, **p<0.01, ****p<0.0001 were calculated using the Student’s t-test. (D–F) Quantitative analysis of the trabecular parameters of the distal tibia of control mice and model mice treated with vehicle or benzbromarone. Tb.N: trabecular number (D); Tb.Th: trabecular thickness (E); Tb.Sp: trabecular separation (F). Arrows indicate joint damage and bone erosion. Results are shown as the mean±SD (n=6 mice per group). The data were analysed by unpaired two-tailed Student’s t-test, *p<0.05, **p<0.01, ****p<0.0001.

To validate the clinical assessments, the severity of bone and cartilage erosion in the hind paws of the representative control group, the model mice and the model mice treated with benzbromarone were assessed by micro-CT and X-ray imaging. As shown in figure 2A, the three-dimensional images of bones in the hind paws were reconstructed. Moderate bone erosion was observed in model mice at 2 months after the initial induction of gouty arthritis. By month 4, when compared with control group, bone erosion, damage, deformity, bone surface roughness and joint destruction were found to be increasingly serious in model mice following the continuous induction (figure 2A). However, less bone erosion and deformity were observed in benzbromarone-treated model mice in comparison with untreated mice. The semiquantification of bone erosion appears to be in agreement with subjective visual examination of micro-CT images (figure 2C).

Micro-CT measurements on the hind paws and ankle joints revealed that the model mice definitely developed bone erosion and experienced severe bone loss compared with the controls (figure 2A–C). The microarchitecture of the distal tibia trabecular bone involved in the ankle joint was analysed by micro-CT, and three-dimensional images of trabecular bone for each group were reconstructured (figure 2B). Compared with the control group, model mice showed a significant decrease in Tb.N (p<0.001 at 4 months after beginning the induction of gouty arthritis) (figure 2D), Tb.Th (p<0.01) (figure 2E) and had a higher Tb.Sp (p<0.01) (figure 2F). Additionally, benzbromarone treatment in model mice progressively attenuated bone erosion and bone loss in comparison with the vehicle-treated model mice (figure 2B–F).

**Figure 3:**
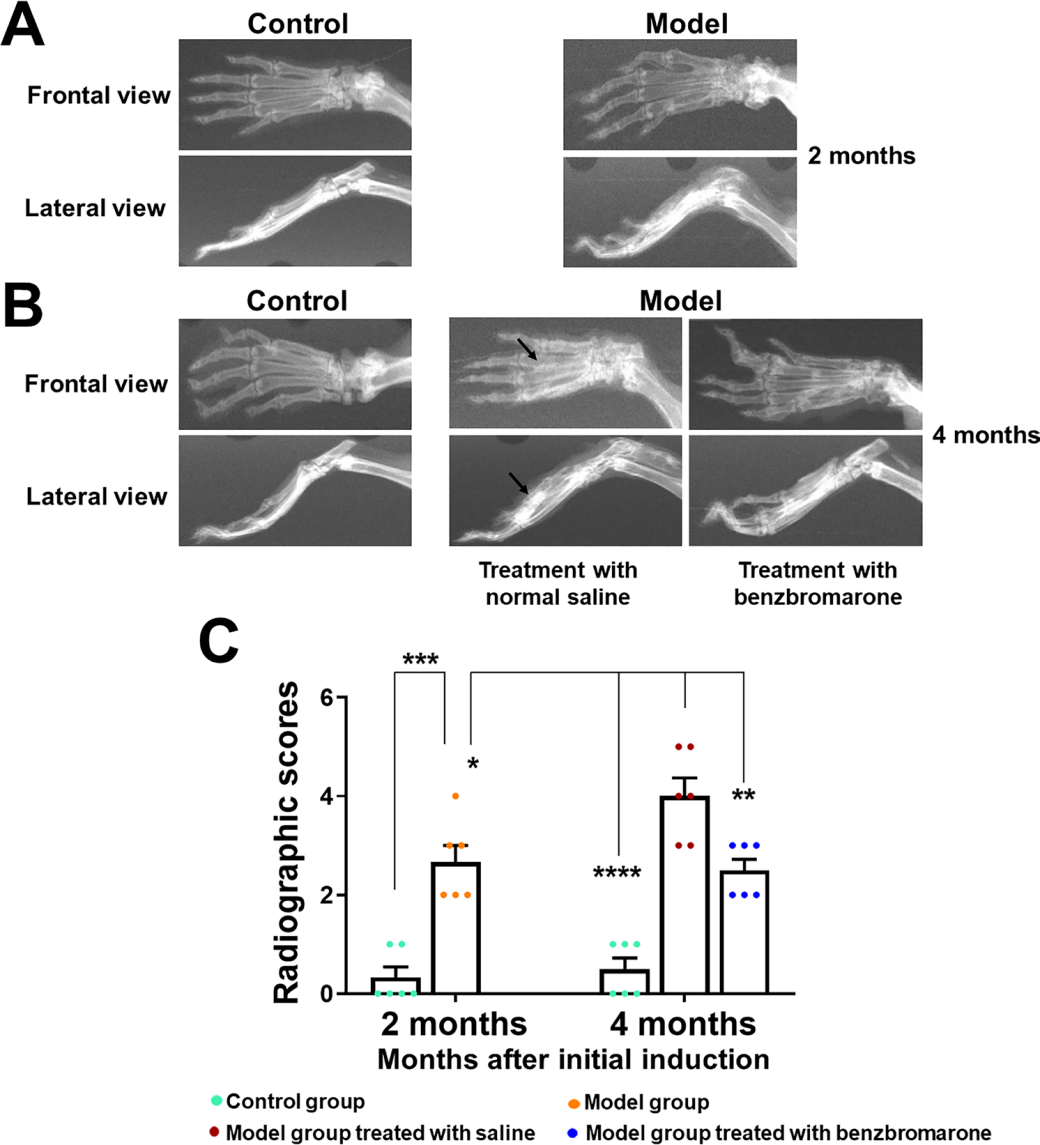
Visualisation of bone erosion and joint damage by X-ray imaging. (A) Frontal and lateral radiographs of the hind paws obtained from normal control mice and hyperuricaemia-induced gouty arthritis model mice at 2 months after the initial induction. (B) Frontal and lateral X-ray pictures of representative paws from naïve and treated or untreated gouty arthritis mice. Gouty arthritis mice were treated with benzbromarone or vehicle for 2 months, starting at 2 months after the initial induction. (C) X-ray scores for the destruction of bones and joints of hind paws were assessed. Arrows indicate joint destruction, bone surface roughness as well as fuzzy and narrow paw joints. Results are shown as the mean±SD for 6 animals in each group. *p<0.05, **p<0.01, ***p<0.001, ****p<0.0001 were calculated using the Student’s t-test.

To assess the degree of bone erosion and joint damage, X-ray images of the hind paws were collected at 2 and 4 months after the initial induction. The plain radiographs in the control group showed obviously preserved architecture of paw bones and ankle joints. The structure in the lateral view and frontal view was complete, the joint space was clear and bone surface was smooth. In the model and vehicle-treated groups, the bones displayed signs of apparent erosion and degradation, the ankle and paw joints were fuzzy and narrow (figure 3A,B). In contrast, benzbromarone treatment significantly reduced the radiographic signs of gouty arthritis in the model mice (figure 3B,C), and X-ray scores were significantly lower in the benzbromarone-treated mice (p<0.05) (figure 3C).

### Histological analysis

**Figure 4:**
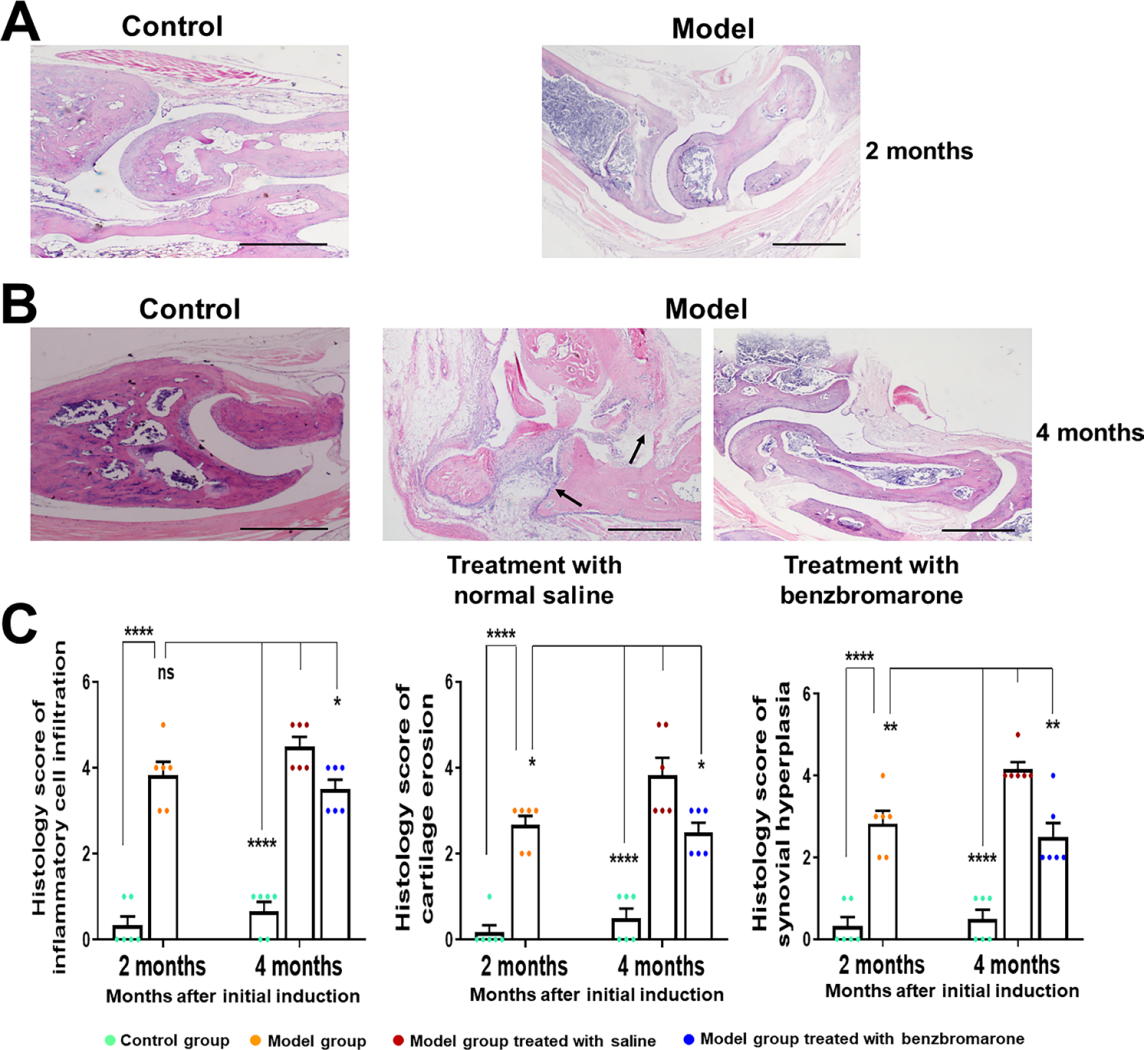
Histological analysis of gouty arthritis model mice. Histological analyses were performed on paw joints stained by H&E. Inflammatory cell infiltration, cartilage erosion and synovial hyperplasia were observed in model mice at both 2 (A) and 4 (B) months after the initial induction. (C) The mean score of inflammatory cell infiltration, cartilage erosion and synovial hyperplasia were calculated using a scale of 0–5 (0=normal; 1=minimal; 2=mild; 3=moderate; 4=marked; 5=severe). Arrows indicate synovial hyperplasia and inflammatory cell infiltration. Data are represented as the mean±SD for 6 mice in each group. *p<0.05, **p<0.01, ****p<0.0001 were calculated using the Student’s t-test. The scale bar represents 500 µm.

Gouty arthritis is characterised by inflammatory cell infiltration, bone erosion and synovial hyperplasia in the affected joints. To assess the histological outcome in the model of gouty arthritis, hind paws were fixed, sectioned and stained with H&E. Histological sections of ankle joints revealed marked arthritic properties including inflammatory cell infiltration, synovial hyperplasia, bone erosion and cartilage damage in the model group at 2 months (figure 4A) and 4 months (figure 4B) after initial induction of gouty arthritis. These arthritic properties were not observed in the normal mice and were significantly decreased in the group treated with benzbromarone compared with the vehicle-treated group (figure 4C).

**Figure 5:**
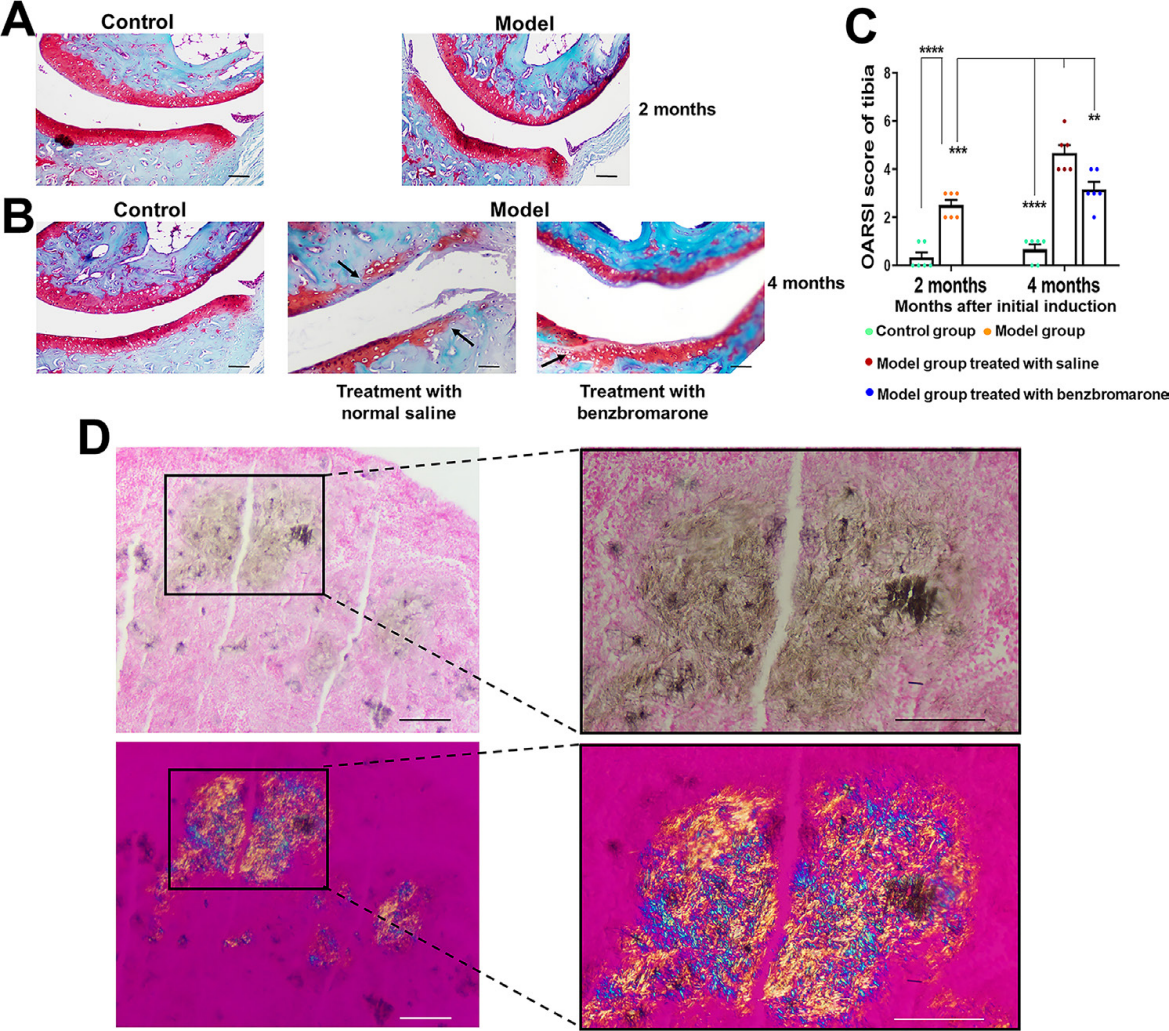
Safranin O-Fast Green and Gomori’s methenamine silver staining analysis. Safranin O-Fast Green staining analysis for the cartilage degradation of paw joints was performed at 2 (A) and 4 months (B) after induction of gouty arthritis. Arrows indicate cartilage destruction and roughness of the articular cartilage surface. (C) OARSI scores are represented as the mean±SD (n=6 for each group). **p<0.01, ***p<0.001, ****p<0.0001 were calculated using the Student’s t-test. (D) Representative Gomori’s methenamine silver staining of MSU crystal deposition in synovium from model group at 4 months after gouty arthritis induction, and viewed under light microscope and polarised light, respectively. The scale bar represents 50 µm. MSU, monosodium urate; OARSI, Osteoarthritis Research Society International.

The cartilage damage in the paw joints of gouty arthritis model mice was further analysed by Safranin O-Fast Green staining which was used to evaluate the loss of proteoglycans in the articular cartilage. As shown in figure 5A–C, the representative Safranin O-Fast Green staining images of paw joints of normal mice indicated that the cartilage surfaces were flat and smooth, the cartilage structure was clear and complete, and that there were no chondrocytes clustering or inflammatory cell infiltration. In the model group, severe cartilage destruction was observed, the surface of the articular cartilage was rough and the intensity of Safranin-O staining was reduced, however, treatment with benzbromarone could significantly alleviate the cartilage damage and rescue the proteoglycan contents in the articular cartilage (figure 5B).

Additionally, the OARSI scores which are used to evaluate the severity of distal tibial articular cartilage showed that the severity in model mice was greater than that in normal mice, and benzbromarone treatment group had markedly lower OARSI Scores than that in vehicle treatment group (figure 5C).

Furthermore, the macroscopic appearance of MSU crystal deposition or tophi was observed in the paw joints or ankle joints of 37.9% of model mice (11 out of 29 mice) at 4 months after initial induction. The MSU crystal deposition was confirmed by Gomori’s methenamine silver stain. The crystals appeared as yellow-brown aggregates of the needle-shaped structures and birefringent structures under light microscope and polarising microscope, respectively (figure 5D).

### The production of proinflammatory mediators in the hind paw tissues of hyperuricaemia-induced gout mouse model

**Figure 6:**
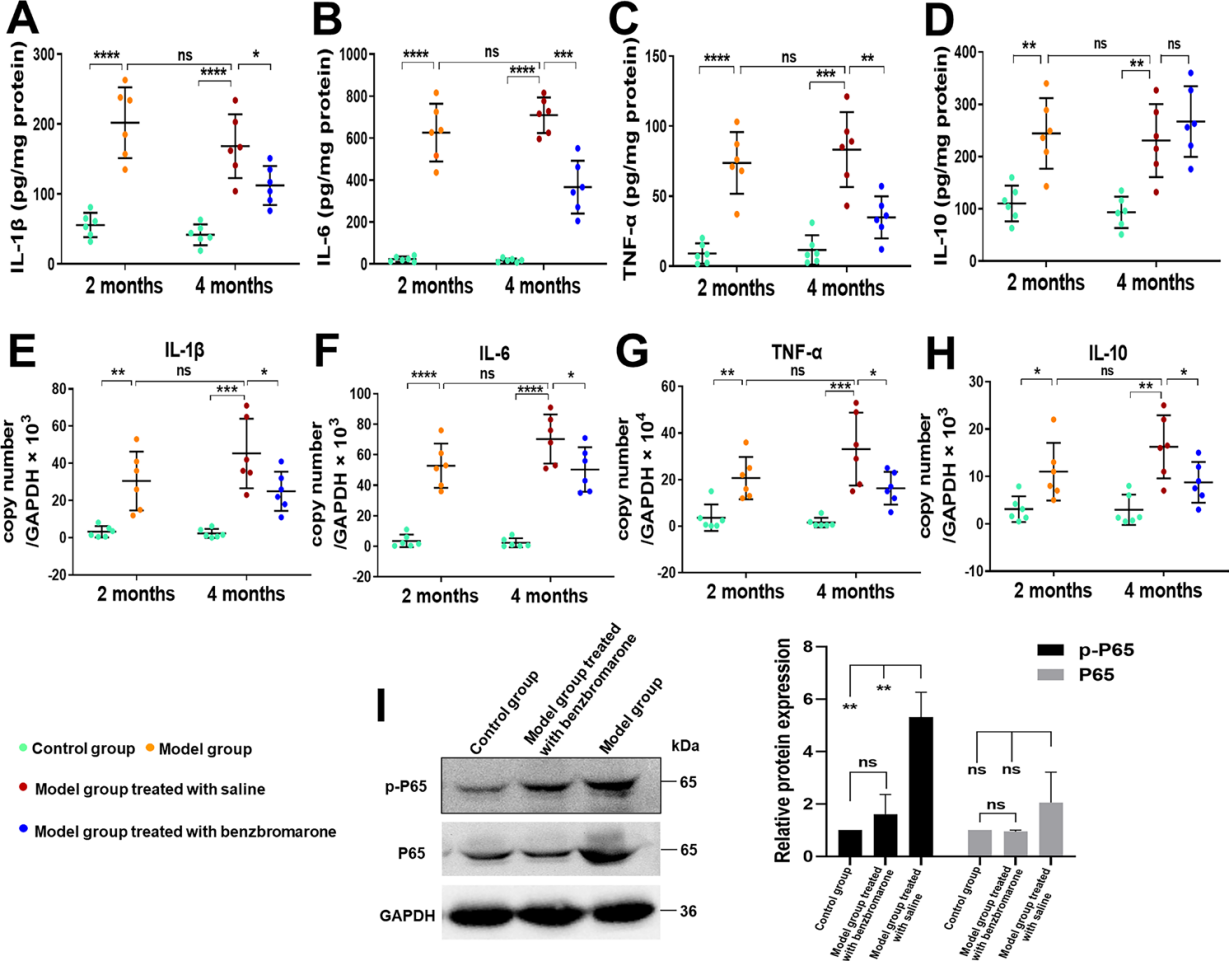
Production of inflammatory cytokines analysed by ELISA and qRT-PCR in arthritic paws. The concentrations of IL-1β (A), IL-6 (B), TNF-α (C) and IL-10 (D) in the supernatants of paw homogenates obtained from each group of animals were measured by ELISA. The values were normalised with total protein concentration in the supernatants. (E–H) qRT-PCR analysis of the indicated mRNA levels in the arthritic paws. The mRNA levels of IL-1β (E), IL-6 (F), TNF-α (G) and IL-10 (H) are expressed relative to the GAPDH mRNAs. Results are represented as the mean±SD (n=6 for each group). *p<0.05, **p<0.01, ***p<0.001, ****p<0.0001 were calculated using the Student’s t-test. (I) The phosphorylation and expression levels of NF-κB p65 in the homogenates of arthritic paws obtained from model mice at 4 months after induction were assessed *via* Western blot, with GAPDH as a loading control. Images presented are representative of three independent experiments, and data are expressed as the mean±SD. The significance was determined by the Student’s t-test and *p<0.05, **p<0.01, ***p<0.001 and ns (not significant) as compared with the controls. GAPDH: glyceraldehyde-3-phosphate dehydrogenase.

ELISA and qRT-PCR were used to detect the levels of cytokines TNF-α, IL-1β, IL-6 and IL-10 in the hind paw tissues. When compared with the control group, the protein levels of the proinflammatory mediators (TNF-α, IL-1β and IL-6) and the anti-inflammatory factor IL-10 examined were significantly elevated in the paw tissues of model mice at both 2 months and 4 months after the initial induction of gouty arthritis (figure 6A–D), while no significant differences were viewed in TNF-α, IL-1β, IL-6 and IL-10 protein levels between model mice at 2 months and model mice 4 months after initial induction of gouty arthritis. Benzbromarone treatment substantially decreased the expression of cytokines TNF-α, IL-1β, IL-6 and IL-10. Changes in the mRNA levels of cytokines in hind paw tissues and the effects of benzbromarone on reducing the mRNA expression of these four cytokines were further confirmed using qRT-PCR (figure 6E–H). To explore the mechanistic basis for the production of inflammatory cytokines in an arthritic paws, we assessed the phosphorylation and expression levels of NF-κB P65 in the hind paw tissue homogenates of gouty arthritis mice at 4 months after induction. This analysis revealed that hyperuricaemia-induced gout (HIG) model mice had higher expression and phosphorylation levels of NF-κB P65 as compared with control group. In contrast, benzbromarone treatment markedly inhibited P65 expression and phosphorylation (figure 6I).

**Figure 7:**
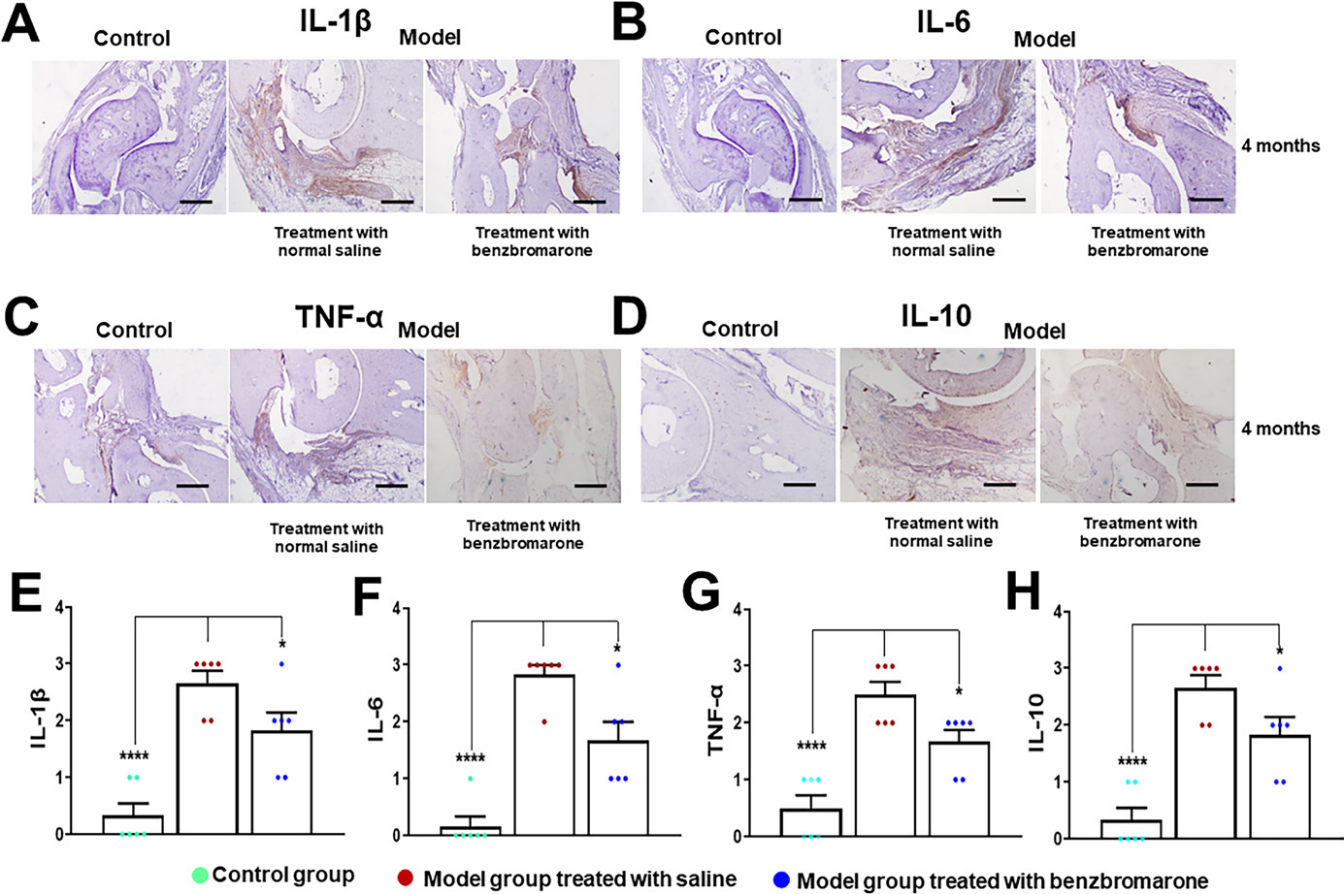
Immunohistochemical staining for IL-1β, IL-6, TNF-α and IL-10 in the mouse model of gouty arthritis. Immunohistochemical analysis of paw joints obtained from model mice at 4 months after induction showed markedly positive staining for IL-1β (A), IL-6 (B), TNF-α (C) and IL-10 (D). In contrast, the positive staining reactions for IL-1β, IL-6, TNF-α and IL-10 were significantly decreased in model group treated with benzbromarone and were almost undetectable in control group. Semiquantitative analysis of immunohistochemical expression levels of IL-1β (E), IL-6 (F), TNF-α (G) and IL-10 (H) in arthritic paws. Data are represented as the mean±SD (n=6 for each group). *p<0.05, ****p<0.0001 were calculated using the Student’s t-test. The scale bar represents 200 µm.

Immunohistochemical analysis of paws and joints in The HIG model mice at 4 months after initial induction revealed remarkable expression of the pro-inflammatory cytokines IL-1β (figure 7A), IL-6 (figure 7B), TNF-α (figure 7C) and anti-inflammatory factor IL-10 (figure 7D), while the expression levels for these cytokines were significantly reduced following benzbromarone treatment and almost undetectable in control mice (figure 7A–D).

### The production of reactive oxygen species in the hind paws of HIG model mice

The production of reactive oxygen species is a hallmark of inflammatory processes as well as being related to the progression of gouty arthritis, both neutrophils and macrophages rely on phagocyte NADPH oxidase as the primary source of superoxide production. Therefore, NADPH oxidase activity and extracellular superoxide production were detected by using lucigenin.

In comparison with control mice, the lucigenin bioluminescence was significantly increased in the hind paws of model mice at both 2 months (online supplemental figure S5A) and 4 months (online supplemental figure S5B) after initial induction of gouty arthritis, especially in model mice at 4 months after initial induction (online supplemental figure S5A-C). Notably, model mice treated with benzbromarone exhibited low inflammatory bioluminescence (online supplemental figure S5B, C).

## Discussion

In this study, we have developed a novel mouse model of gout which involves repeated daily injections of a low dose of potassium oxonate to moderately increase the sUA levels, combining this with a high fat-diet and injection of 0.1% acetic acid into the hind paws of mice to facilitate the formation of MSU crystals. Compared with the acute gout model induced by the direct injection of MSU crystals, the chronic mouse model of gout developed in this study more accurately replicates the pathological progression of human gout. This includes the natural formation and deposition of MSU crystals, the chronic and ongoing destruction of bone and joint tissue, cartilage erosion, synovial hyperplasia and the infiltration of inflammatory cells. Hence, this model is better suited for research on the chronic phase of gout, and further investigation is needed to determine whether they can replicate the symptoms and pathological features observed in the acute phase of human gout. Importantly, benzbromarone, a drug promoting the excretion of UA, could prevent progressive crystal deposition and tophi formation, reverse articular inflammation and improve the signs and symptoms of gout in mice of model group. Additionally, the plasma glucose levels of model mice were significantly elevated at 2 months and 4 months after the initial induction of gouty arthritis, while no significant body weight changes and no obvious renal pathological damage was observed in model mice. This study provides a preclinical model to test therapeutics and will be instrumental in further exploring the molecular and cellular mechanisms underlying gouty arthritis.

Gout is the most prevalent form of inflammatory arthritis and is induced by the deposition of MSU crystals in multiple joints and soft tissues.^1 23^ Currently, various animal models of gout have been established, primarily induced by the direct injection of MSU crystals into articular locations or air pouches. These models efficiently reproduce the acute inflammatory response observed during human acute gouty attacks, highlighting their value in studying this aspect of the disease.^15^ However, they only partially reflect the complexity of gout as a chronic disease characterised by MSU crystal deposition. Notably, these models do not capture the features of chronic gouty arthritis, including the development of chronic tophaceous gout during disease progression.^24 25^

Hyperuricaemia is recognised as the most important risk factor for the development of gout.^25^ To our knowledge, no mouse model with hyperuricaemia established using potassium oxonate, a competitive uricase inhibitor, has developed gouty arthritis. One reason for this is that a persistently high level of sUA leads to severe hepatic and renal dysfunction and mouse death before the development of gouty arthritis.^26–28^ A low level of sUA is not sufficient to induce gouty arthritis, given the short lifespan of mice and that MSU crystal growth requires sufficient urate load.^25^ Therefore, we moderately increased the sUA levels by administering a low dose of potassium oxycyanide (online supplemental figure S1B), with the aim of averting any potential harm to liver and kidney functions resulting from sustained high sUA levels (online supplemental figure S4). Concurrently, we employed other inducing factors to facilitate the formation of MSU crystals in the paw joints.

Besides hyperuricaemia, other known risk factors for the development of gout include genetic factors, high consumption of meat, seafood and alcohol, renal disease, osteoarthritis and metabolic syndrome such as hypertension and obesity.^1 29 30^ It is well known that patients with metabolic syndrome usually have high level of serum urate and hyperuricaemia is recognised as a new marker of metabolic syndrome.^6 31^ Epidemiological studies have demonstrated a close relationship between sUA levels and the development of metabolic syndrome^10 32^ and higher adiposity is a strong risk factor for gout.^33^ Other studies have defined the relationship between insulin resistance and gouty arthritis, and suggested that hyperuricaemia in gout might be caused by the increased adiposity associated with insulin resistance.^34–36^ Based on the above, we fed mice a high-fat diet to promote the formation of MSU crystals, and the model mice exhibited symptoms of metabolic syndrome, including elevated blood glucose levels (online supplemental figure S2).

While the above are clear risk factors for gout, it has also been shown that other local factors play a role in the formation of MSU crystals, such as mechanical stress, cartilage components, synovial fluid and pH values.^4^ Growing evidence indicates that there is a link between cartilage damage and MSU crystal formation.^4 37^ Recently, an interesting study has shown that human cartilage homogenates increase MSU crystal formation and promote the formation of smaller crystals in the presence of high urate concentrations. It has been suggested that small crystals have a higher inflammatory potential.^5^

**Figure 8:**
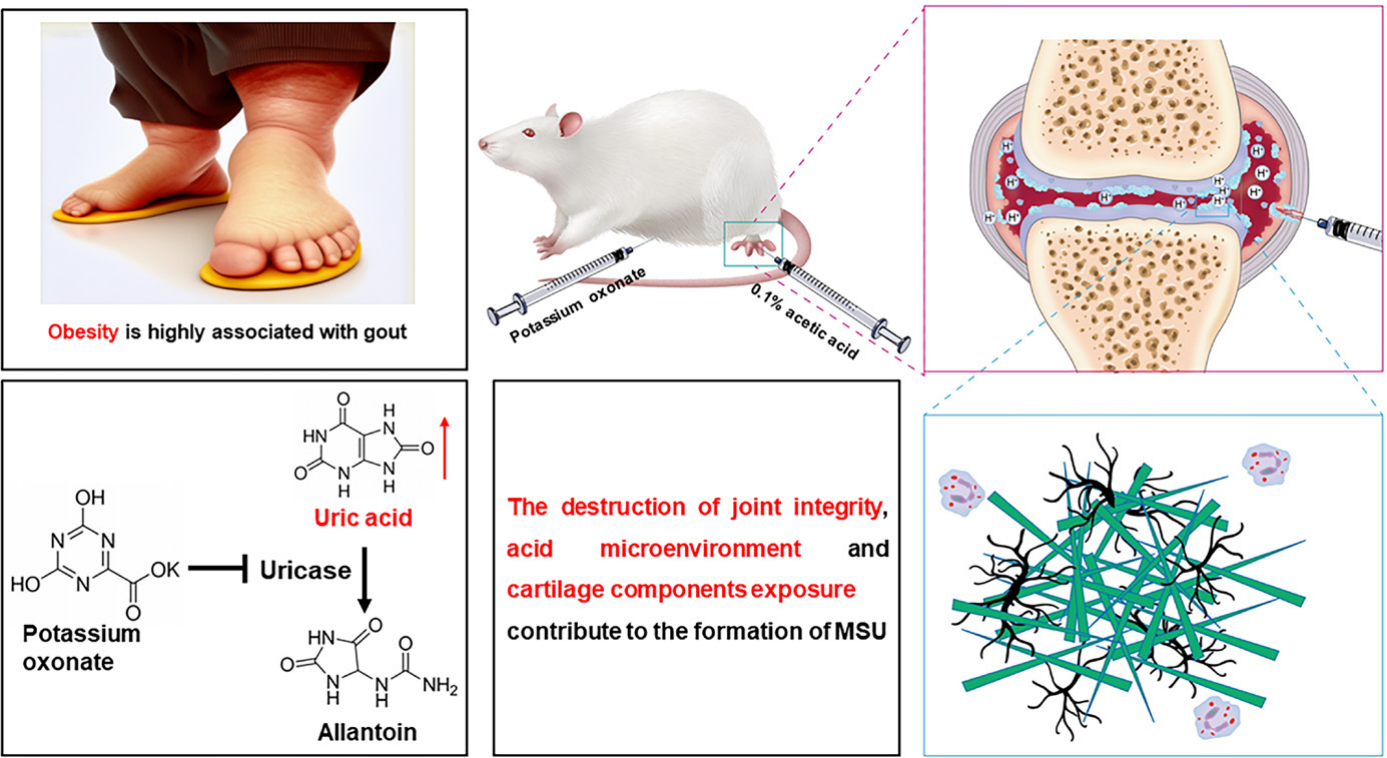
Schematic representation of the methods employed to establish a chronic mouse model of gout. A novel chronic mouse model of gout is established by administering daily intraperitoneal injections of a low dose of potassium oxonate for approximately 4 months, along with a high fat-diet and injections of 0.1% acetic acid into the hind paws of the mice. Intraperitoneal injection of low-dose potassium oxonate moderately increases the level of serum uric acid in mice and prevents kidney and liver injuries caused by sustained high levels of uric acid. Obesity is closely associated with the occurrence of gout. The injection of acetic acid into the hind paw not only compromises joint integrity but also creates an acidic microenvironment and exposes the cartilage components. The destruction of joint integrity, acid microenvironment and cartilage components exposure may contribute to the formation of MSU. MSU, monosodium urate.

Like cartilage components, the presence of an acid microenvironment also facilitates the formation of MSU crystals in an in vitro system.^4^ Consistent with this phenomenon, alcohol overconsumption and respiratory insufficiency usually leads to acidosis as well as acute gout attacks, since systemic acidosis inhibits renal UA excretion resulting in hyperuricaemia.^4^ In addition, increased level of lactic acid and a lower pH have been observed in the synovial fluid from patients with acute gout (figure 8).^4^ Therefore, the injection of acetic acid applied in this study not only leads to the disruption of joint integrity and the exposure of cartilage components but also results in the formation of an acidic microenvironment within the joint. Additionally, it induces aseptic inflammatory responses. All of these factors work together to facilitate the formation and deposition of MSU crystals in the paw joints.

There are several limitations of the current study. First, more time (2–4 months) will be taken to establish the chronic mouse model of gouty arthritis in comparison with the mouse model of MSU crystal-induced acute gouty arthritis. Second, the deposition of urate crystals in the hind paws of a small sample size of mice (n=8) was detected by Gomori’s methenamine silver stain. Further studies with an enlarged sample size will contribute to comprehensively evaluate the deposition of MSU crystals in model mice as well as its correlation with the clinical phenotype such as paw swelling and redness. Third, to simplify experimental design or else more mice will be involved in this study, we only observed the combined effects of a high fat-diet and the injection of o.1% acetic acid into the hind paws on the HIG arthritis without considering their respective effects.

In conclusion, we successfully developed a chronic mouse model of gout by increasing the levels of sUA, lowering the microenvironment pH of paw joints and implementing a high-fat diet. The resulting chronic mouse model accurately replicated the clinical and pathological features observed in human gout, making it highly suitable for further investigations into the pathogenesis of gouty arthritis. Moreover, this model holds great potential as an ideal screening platform for testing antigout agents.

## Data Availability

No data are available.
