# Supplementary material for: New animal model of chronic gout reproduces pathological features of the disease in humans
Source: RMD Open. 2023 Nov 16;9(4):e003499. doi: 10.1136/rmdopen-2023-003499 (PMC10660916; doi:10.1136/rmdopen-2023-003499)
Supplement: Supplementary data [file rmdopen-2023-003499supp001.pdf]

Supplementary materials

A chronic mouse model of gout reproduces the pathological features of human gout

Jiwei Wang,[1,2†] Peiqi Hao,[1,2†] Xianrun Sun,[3†] Richard Ward,[4] Tao Tang,[5] Xi Chen,[2] Yihong Liu,[2] Guancong Luo,[2] Yang Yang,[2] Cheng Xiang,[2] Su An,[1,2*] Tian-Rui Xu[1,2*]

[1]State Key Laboratory of Primate Biomedical Research, Kunming University of Science and Technology, Kunming 650500, Yunnan, China;

[2]Faculty of Life Science and Technology, Kunming University of Science and Technology, Kunming 650500, Yunnan, China;

[3]Department of Orthopedic Surgery, The First People's Hospital of Yunnan Province, Affiliated Hospital of Kunming University of Science and Technology, Kunming 650032, Yunnan, China;

[4]Centre for Translational Pharmacology, Institute of Molecular Cell and Systems Biology, College of Medical, Veterinary and Life Sciences, University of Glasgow, Glasgow G12 8QQ, UK;

[5]Medical School, Kunming University of Science and Technology, Kunming 650500, Yunnan, China

†These authors contributed equally to this paper.

*** Correspondence:**

Dr. Su An; aslxj@mail.ustc.edu.cn; Dr. Tian-Rui Xu; tianruixu@kust.edu.cn

Supplemental material: Immunohistochemical analysis, cytokine enzyme linked immunosorbent assay (ELISA), quantitative real-time PCR, western blot analysis and in vivo lucigenin bioluminescence imaging of NADPH oxidase activity.

Immunohistochemical analysis

For immunochemistry, hind paws were fixed in 10% neutral buffered formalin, decalcified by 14% EDTA, embedded in paraffin wax and sectioned. Subsequently paraffin wax was removed and the sections rehydrated with xylene and ethanol, respectively. Endogenous peroxidase was blocked with 3% hydrogen peroxide. Antigen retrieval was performed using 10 mM citrate buffer (pH 6.0) for 10 s at 100 ℃, then slides were cooled and rinsed in distilled water and Tris-buffered saline with Tween 20 (TBST). After blocking with 5% normal goat serum/TBST (Cell Signaling Technology, Cambridge, USA #5425), the following primary antibodies were used: anti-IL-1β (mouse mAb, diluted 1:100, Cell signaling Technology, Cambridge, USA #12242), anti-IL-6 (rabbit pAb, diluted 1:200, Abcam, Cambridge, UK #ab7737), anti-IL-10 (rat mAb, diluted 1:100, Santa Cruz Biotechnology, Santa Cruz, CA). and anti-TNF-α (mouse mAb, diluted 1:100, Santa Cruz Biotechnology, Santa Cruz, CA). After incubating with primary antibody overnight at 4 ℃, the sections were treated with Signalstain Boost IHC detection reagent (Cell Signaling Technology, Cambridge, USA. HRP, mouse #8125; HRP, rabbit, #8114) for 30 min at room temperature, and visualized using the Signalstain DAB substrate kit (Cell Signaling Technology, Cambridge, USA #8059) for 1 to 10 min, followed by counterstaining with hematoxylin. After

Wang J, *et al. RMD Open* 2023; 9:e003499. doi: 10.1136/rmdopen-2023-003499

immunohistochemical staining, the expression of different inflammatory factors in the synovial tissue of paw and ankle joints was scored semi-quantitatively on a four-point scale independently and blindly by two individual pathologists, and the average of their scores was calculated. A score of 0 represented minimal expression; 1, mild expression; and 2, moderate expression; whereas 3 represented abundant expression of inflammatory factors.

Cytokine enzyme linked immunosorbent assay (ELISA)

The ankle joints and paws of mice were dissected, weighed and placed into cold phosphate buffered saline (PBS) containing protease inhibitor cocktail (Roche Diagnostics) at a final concentration as recommended by the manufacturer, then homogenized with a QM100 bead miller (Wuzhou Ding Chong, Beijing, China) for 2 min at room temperature. The homogenates were centrifuged twice at 15,000 r.p.m for 20 min at 4 ℃ to remove tissue and cell debris and the resulting supernatants were aliquoted and stored at -80 ℃. The pro-inflammatory cytokines (TNF-α, IL-1β and IL-6) and anti-inflammatory cytokine (IL-10) in the supernatants were measured using Abcam's ELISA kit (Abcam, Cambridge, UK), according to the manufacturer's instructions. Briefly, diluted samples or standards were added to 96-well plated pre-coated with affinity purified antibody specific for mouse TNF-α, IL-1β, IL-6 and IL-10, respectively. The wells were washed and biotinylated anti-mouse TNF-α, IL-1β, IL-6 and IL-10 antibodies were added as appropriate. After washing away unbound biotinylated antibodies, horseradish peroxidase (HRP)-conjugated streptavidin was

pipetted to the wells and tetramethylbenzidine (TMB) was used as a substrate. The intensities of the color detected at 450 nm reflected the level of cytokines present.

Quantitative real-time PCR

Total RNA was extracted from hind paws and ankle joints using RNApre pure Tissue Purification kit (Tiangen, China) following the manufacturer's instructions. First-strand cDNA was synthesized by reverse transcription of the total RNA using the transcript II RT kit (Taingen, China). The cDNA products and corresponding primers were used for iQ SYBR Green Supermix-quantitative real-time PCR (Bio-rad, California, USA) to assay the levels of TNF-α, IL-1β, IL-6 and IL-10. The primers were designed based on the mRNA sequences in GenBank and synthesized by Shanghai Shenggong Botechnology (Shanghai, China). Quantitative RT-PCR was carried out on a Lightcycler96 detection system (Roche, Basel, Switzerland) with mouse β-actin as an internal control. The fold change in the gene expression levels of target genes were calculated with values for normalization to GAPDHusing the $2^{-\Delta\Delta Ct}$ comparative cycle threshold method. Amplification was performed in the following cycling conditions: 94 ℃ for 15 s, annealing at 60 ℃ for 30 s, and extension at 72 ℃ for 30 s with a total of 40 cycles. Primer sequences were summarized in online supplemental Table S1.

Western blot analysis

Protein samples were prepared in the same way as for ELISA analysis, and 30 μg protein was loaded on each lane of a 10% SDS-PAGE gel. Following separation,

proteins were transferred to polyvinylidene difluoride (PVDF) membrane (Millipore, MA, USA). The membrane was blocked with 5% non-fat milk in Tris buffer for 2 h at room temperature and incubated overnight at 4 ℃ with the following specific primary antibodies. Monoclonal rabbit anti-NF-κB P65 (catalog no. 59674, 1:1000) and anti-p-NF-κB P65 (catalog no. 3033, 1:1000) were purchased from Cell Signaling Technology (Beverly, MA, USA). Monoclonal rabbit anti-GAPDH was from Abcam (Cambridge, UK #AB181602, 1:10000). The secondary antibody, anti-rabbit IgG, HRP-conjugated antibody was from Cell Signaling Technology (Cambridge, USA #7074, 1:5000). Protein bands were detected using GeneGnome Chemiluminescence Imaging Systems (SYNGENE Bio Imaging, Cambridge, UK).

In vivo lucigenin bioluminescence imaging of NADPH oxidase activity

Lucigenin (bis-N-methylacridinium nitrate) was used to detect NADPH oxidase activity and extracellular superoxide production, as both neutrophils and macrophages rely on phagocyte NADPH oxidase as the primary source of superoxide production. Lucigenin (Sigma Chemical Co., St. Louis, MO, USA #M8010) was dissolved in phosphate buffered saline to make a 2.5 mg/ml stock solution. The stock solution was intraperitoneally injected into mice to achieve final dosage of 25 mg/kg body weight of lucigenin. Bioluminescence imaging was performed on an IVIS lumina II (PerkinElmer) under compressed air containing 2% of isoflurane 20 min following i.p. injection of lucigenin. Acquisition time was 60 seconds, F-stop=1, Binning=8, fovD, and 0 second delay. Bioluminescence was expressed as total radiance

(photons/seconds/cm^2/steradian) and data were analyzed using Living Image software (PerkinElmer).

Supplemental figures

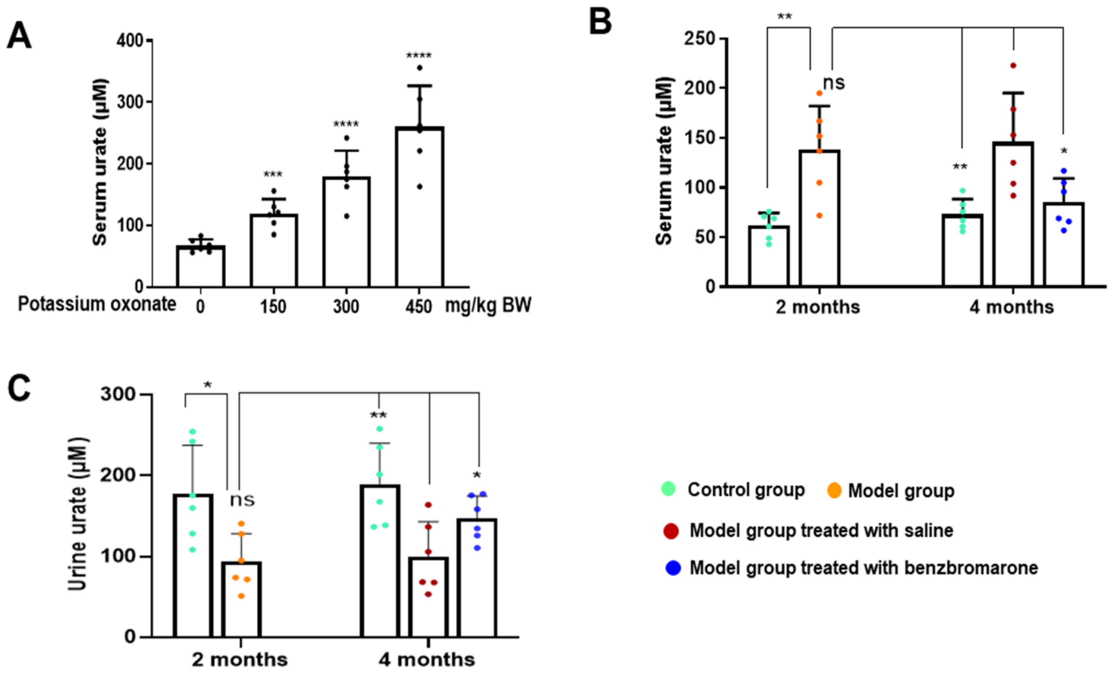

Supplemental figure S1 Serum urate levels in control and model mice. (A) The effects of potassium oxonate concentrations on serum urate levels. (B) The serum urate concentrations of control and model mice were measured at 2 and 4 months after initial induction. (C) 24-hour uric acid levels in the urine of the control and model mice were measured at 2 and 4 months after initial induction. Results are represented as the mean ± SD (n = 6 for each group). *p < 0.05, **p < 0.01, ***p < 0.001 and ****p < 0.0001were calculated using the student's t-test.

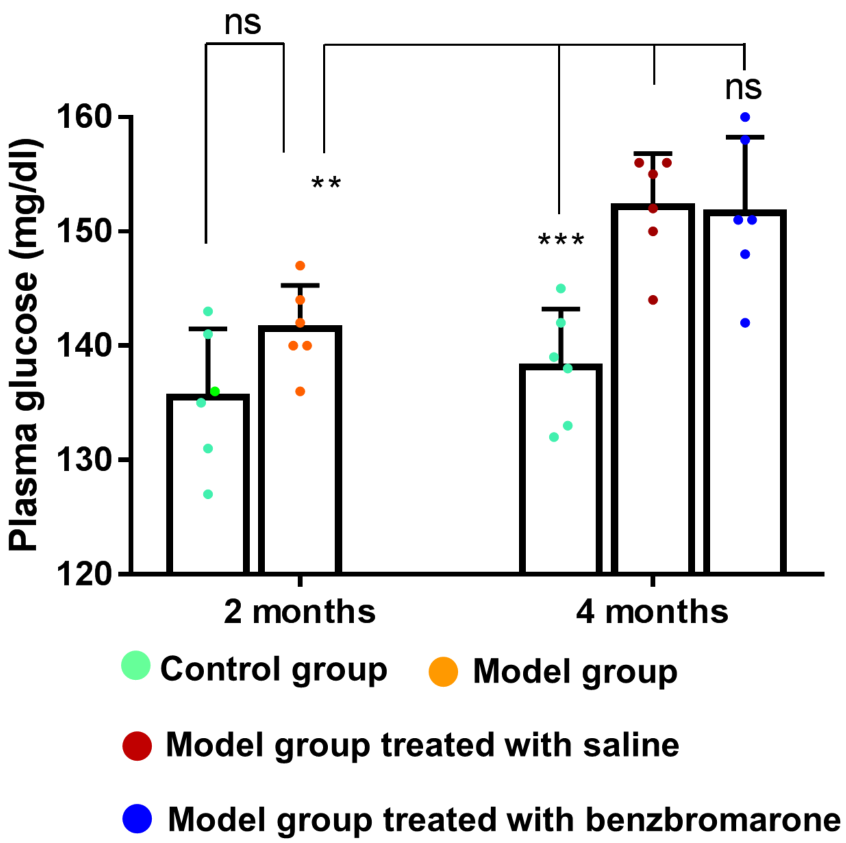

Supplemental figure S2 Plasma glucose levels in control and model mice. The plasma glucose concentrations of control and model mice were measured at 2 and 4 months after initial induction. Results are represented as the mean ± SD (n = 6 for each group). **$p < 0.01$ and ***$p < 0.001$ were calculated using the student's *t*-test.

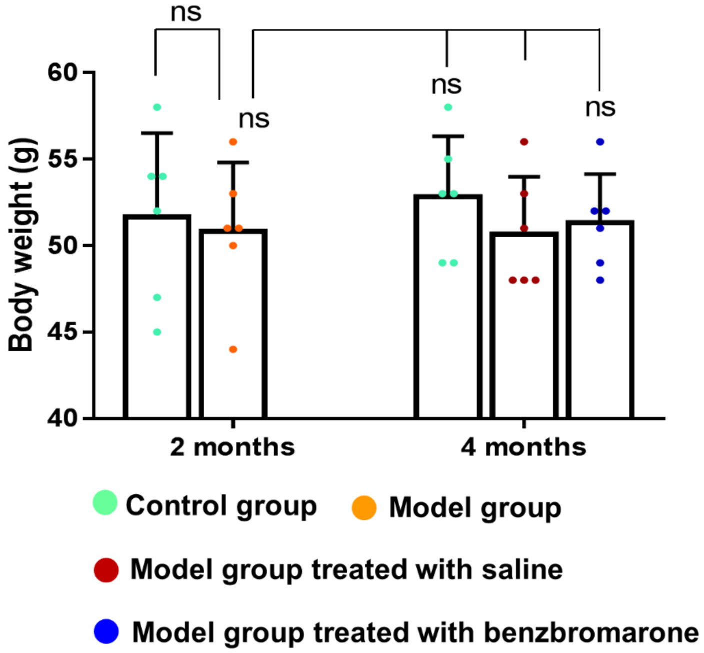

Supplemental figure S3 Body weight changes of control and model mice. The body weight changes were investigated in the control and model mice at 2 and 4 months after initial induction. Results are represented as the mean ± SD (n = 6 for each group). ns means not significant.

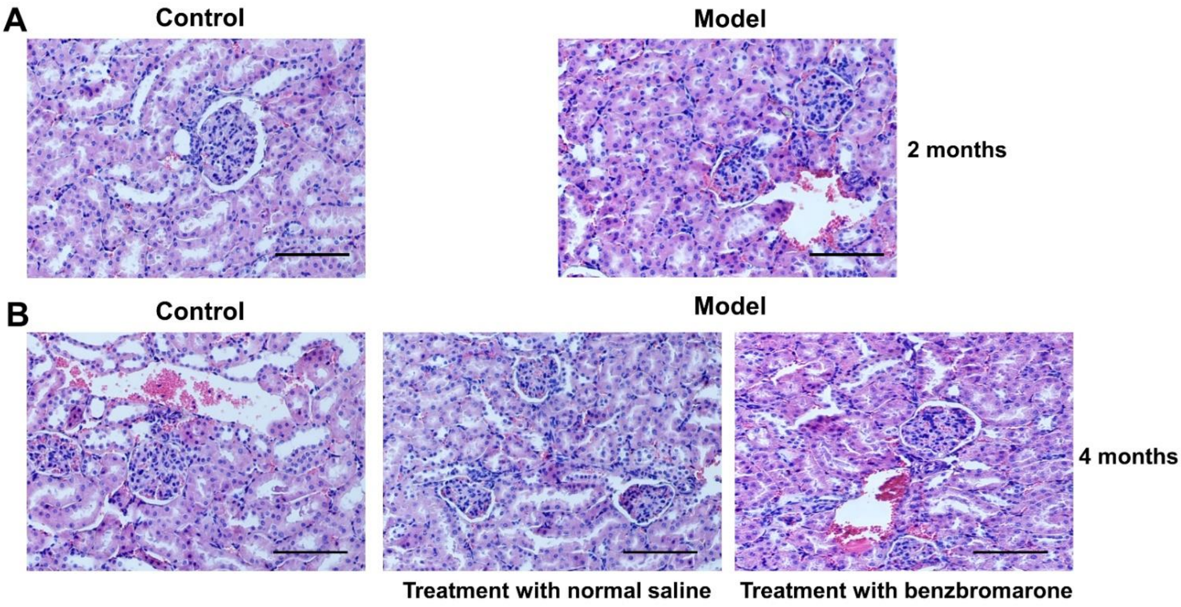

Supplemental figure S4 Representative images of renal pathological changes in gouty arthritis model mice. Histological analyses were performed on renal tissues stained by haematoxylin and eosin. The damage of renal glomeruli and tubules, renal interstitial fibrosis and inflammatory cell infiltration were analyzed in the control and model mice at 2 (A) and 4 (B) months after initial induction. The scale bar represents 100 µm.

Wang J, *et al. RMD Open* 2023; 9:e003499. doi: 10.1136/rmdopen-2023-003499

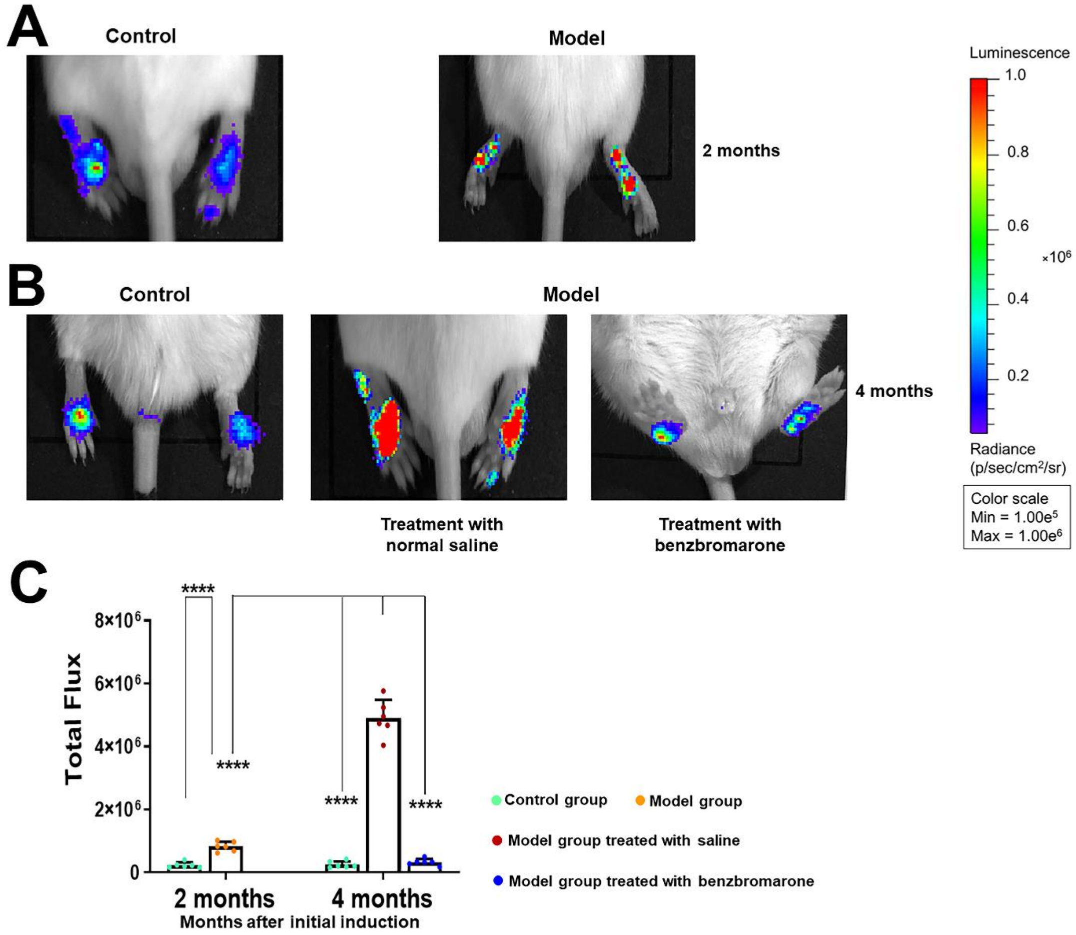

Supplemental figure S5 In vivo imaging of superoxide production in arthritic paws. Representative images of lucigenin-based superoxide detection in arthritic paws of model mice at 2 (A) and 4 months (B) after induction. (C) The quantification of luminescence in the arthritic paws 20 min following i.p. injection of lucigenin (25 mg/kg body weight). Values are the mean ± SD photons/second (p/s) (n = 6 for each group). ****$P < 0.0001$ was calculated using the student's *t*-test.

BMJ Publishing Group Limited (BMJ) disclaims all liability and responsibility arising from any reliance placed on this supplemental material which has been supplied by the author(s)

Supplemental Table S1. Sequences of primer used in qPCR

**Gene symbol**	**Sense (5'-3')**	**Antisense (5'-3')**
IL-1β	TCATGGGATGATGATGATAACCTGCT	CCCATACTTTAGGAAGACACGGATT
IL-6	CTGGTGACAACCACGGCCTTCCCTA	ATGCTTAGGCATAACGCACTAGGTT
TNF-α	GGCAGGTCTACTTTGGAGTCATTGC	ACATTCGAGGCTCCAGTGAATTCGG
IL-10	ACCTGGTAGAAGTGATGCCCCAGGCA	CTATGCAGTTGATGAAGATGTCAAA
β-actin	TGGAATCCTGTGGCATCCATGAAAC	TAAAACGCAGCTCAGTAACAGTCCG

Wang J, *et al. RMD Open* 2023; 9:e003499. doi: 10.1136/rmdopen-2023-003499
